# Supplementary material for: Human caspase-4 detects tetra-acylated LPS and cytosolic Francisella and functions differently from murine caspase-11
Source: Nat Commun. 2018 Jan 16;9:242. doi: 10.1038/s41467-017-02682-y (PMC5770465; doi:10.1038/s41467-017-02682-y)
Supplement: Supplementary file 1 — Supplementary Information [file 41467_2017_2682_MOESM1_ESM.pdf]

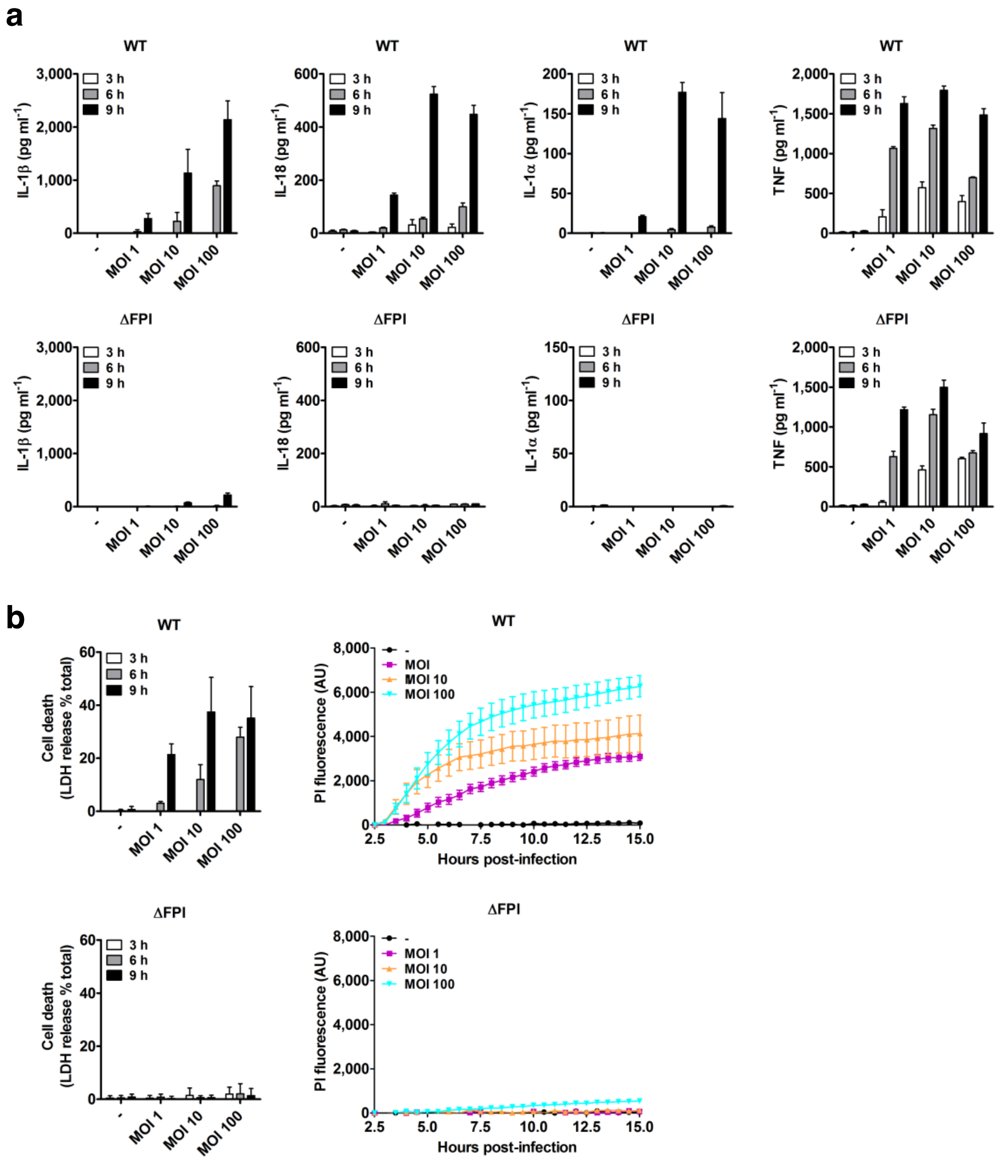

Supplementary Figure 1. ***F. novicida FPI*****-encoded T6SS is required to activate the inflammasome in hMDMs.** (**a,b**) hMDMs were infected with wild-type *F. novicida* (WT) or the ΔFPI mutant at the indicated MOI for the indicated time. (**a**) IL-1β, IL-18, IL-1α, and TNF levels in the supernatant were quantified by ELISA. (**b**) Cell death was assessed by measuring LDH release or propidium iodide (PI) incorporation/fluorescence (AU: arbitrary units). (**a,b**) Mean values ± SD from two to three independent experiments are shown.

a

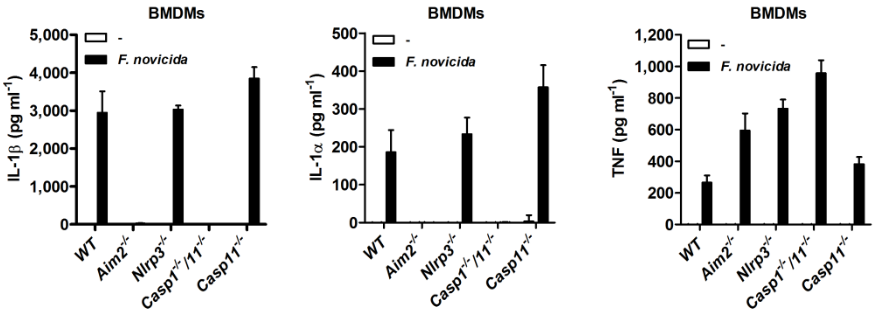

b

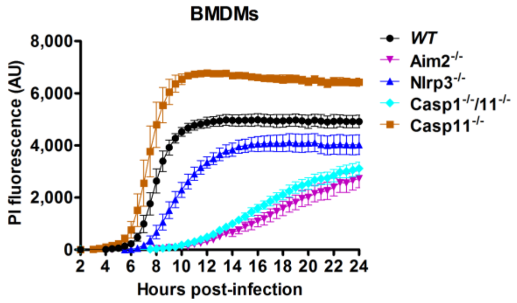

Supplementary Figure 2. AIM2 is the main inflammasome sensor of *F. novicida* in murine macrophages. BMDMs from mice of the indicated genotype were infected with *F. novicida* at a MOI of 10. (**a**) IL-1β, IL-1α and TNF levels in the supernatant were measured by ELISA at 8 h post-infection. (**b**) Real-time cell death was assessed by measuring propidium iodide (PI) incorporation/fluorescence (AU: arbitrary units). (**a,b**) Mean values ± SD from three technical replicates from one experiment representative of three independent experiments are shown.

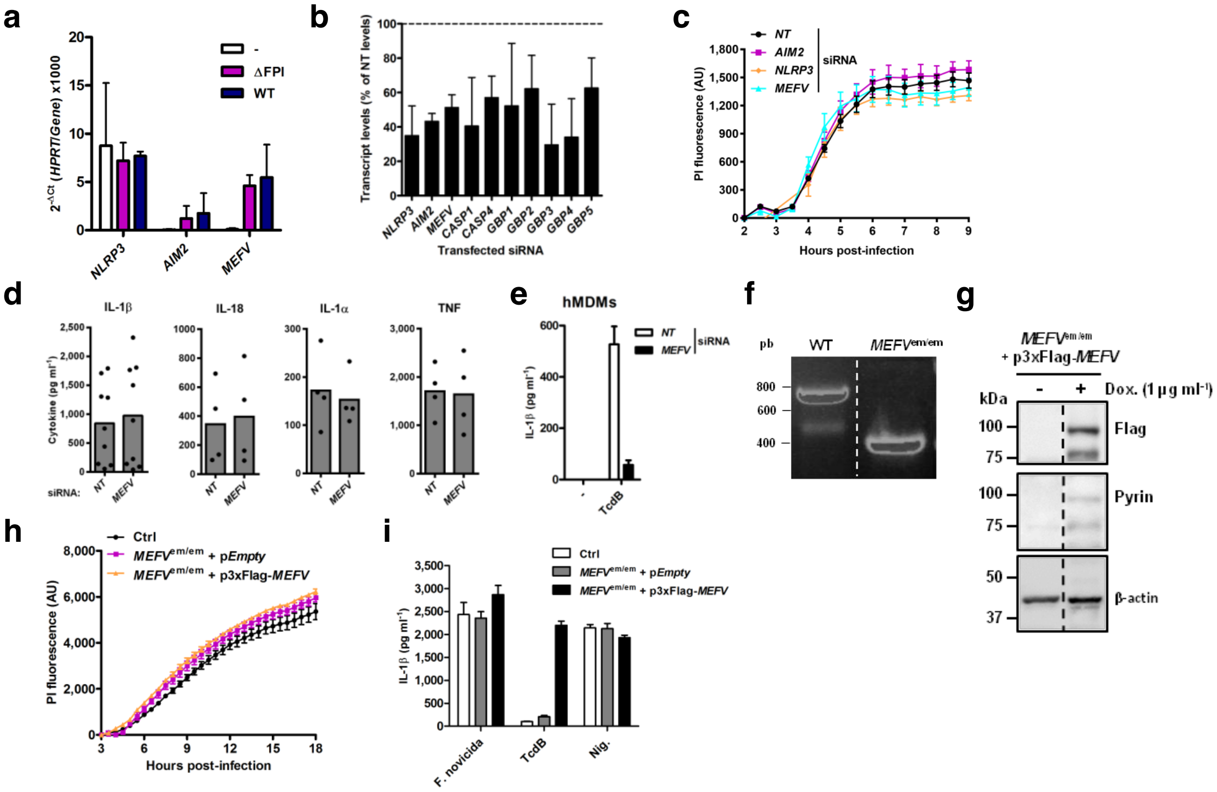

Supplementary Figure 3. Knockdown efficacy; expression of *NLRP3*, *AIM2* and *MEFV* in hMDMs upon infection and effects of AIM2, NLRP3 and MEFV knockdown on inflammasome activation. (**a,b**) qRT-PCR analysis of the indicated transcript levels in hMDMs. (**a**) hMDMs were infected or not (-) with wild-type *F. novicida* (WT) or the ΔFPI mutant at a MOI of 10 for 8 h. (**b**) hMDMs were transfected with non-targeting siRNA (NT) or with the indicated siRNA for 48 h. (**c,d**) hMDMs were transfected with non-targeting siRNA (NT) or with the indicated siRNA before infection with *F. novicida* at a MOI of 10 for 6 h (IL-1β, TNF) or 8 h (IL-18, IL-1α). (**c**) Cell death was measured by assessing every 30 minutes propidium iodide (PI) incorporation/fluorescence (AU: arbitrary units). (**d**) IL-1β, IL-18, IL-1α and TNF levels in the supernatant were measured by ELISA. For each healthy donor, the mean value from cells transfected with NT siRNA was used as a reference value set to 100%. Each point shows the mean value from three technical replicates for one healthy donor. The bar shows the mean for all donors (n ≥4). (**e**) *MEFV* siRNAs knock-down efficacy in hMDMs was validated by functional tests following priming with Pam3CSK4 and *Clostridium difficile* toxin B (TcdB) treatment. IL-1β levels were quantified by ELISA in the supernatant of hMDMs at 3 h post TcdB addition. (**f**) Cas9-expressing U937 cells were transduced with two gRNAs targeting *MEFV* exon 1. Following clonal selection, the endonuclease-mediated (em) gene deletion was validated by PCR. (**g**) Western-blotting analysis using anti-Flag and anti-Pyrin antibodies in U937 *MEFV*$^{em/em}$ cells expressing 3xFlag-*MEFV* after 24 h of treatment with doxycycline. (**h,i**) IFN-γ-treated, PMA-differentiated U937 (Ctrl) and U937 *MEFV*$^{em/em}$ transduced with an empty vector (p*Empty*) or a vector encoding 3xFlag-*MEFV* were infected with *F. novicida* at a MOI of 100 and monitored for inflammasome activation by measuring (**h**) propidium iodide (PI) incorporation/fluorescence in real time (AU: arbitrary units) and (**i**) IL-1β release at 6 h post-infection. (**i**) The functionality and the specificity of Pyrin complementation in U937 *MEFV*$^{em/em}$ cells was validated by measuring IL-1β levels in the supernatant of cell treated with Pam3CSK4 followed by TcdB or Nigericin (Nig.). In agreement with previous work [22], we could not detect Pyrin expression in WT U937 cells. (**a,b**) Mean values ± SD from two independent experiments are shown. (**c**) One experiment representative of three independent experiments with mean values ± SD from three technical replicates is shown. (**e, h** and **i**) Mean values ± SD from three technical replicates from one experiment representative of two independent experiments are shown.

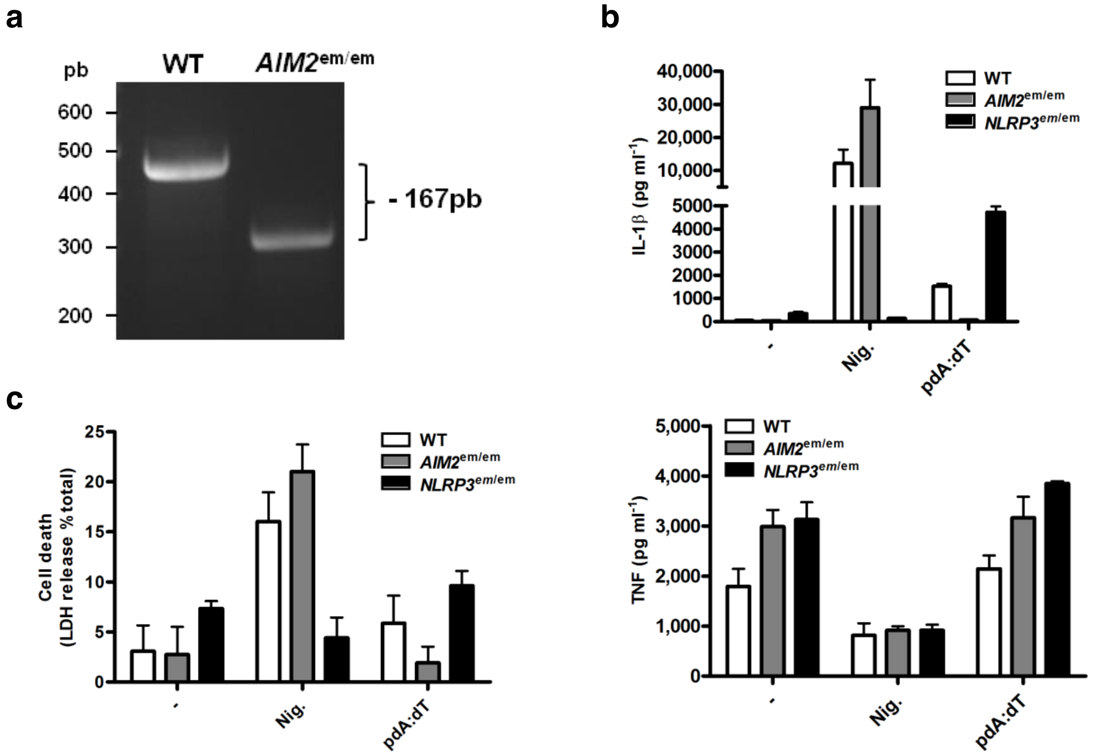

Supplementary Figure 4. Validation of the Crispr/cas9 U937 cell lines. Cas9-expressing U937 cells were transduced with two gRNAs targeting *AIM2* exon 4 or one gRNA targeting *NLRP3* exon 2, respectively. Following clonal selection, the endonuclease-mediated (em) gene deletion was validated by sequencing (not shown); **(a)** by PCR for *AIM2* (in the absence of a specific antibody detecting endogenous human AIM2 level); Western blotting for NLRP3 (see main Fig. 2e); (**b**,**c**) and by functional tests following stimulation with Pam3CSK4 and nigericin (Nig) or poly(dA:dT) transfection. (**b**,**c**) IL-1β, TNF and cell death levels in the supernatant were measured by ELISA or LDH assay, respectively at 2 h (Nig.) or 3 h (pdA:dT) post-treatment. One experiment representative of three independent experiments is shown. Mean values ± SD from three technical replicates are shown.

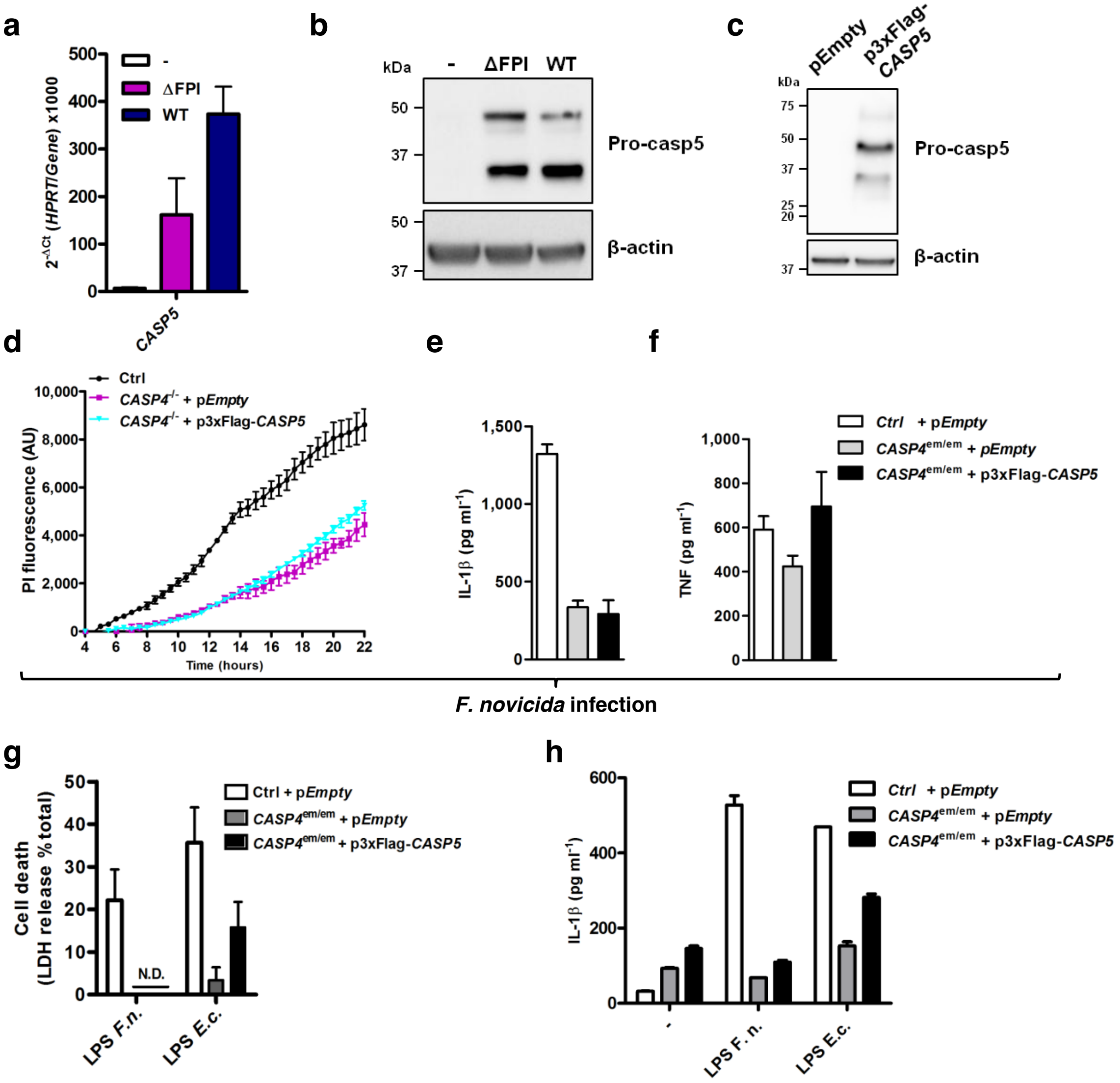

Supplementary Figure 5. No role for caspase-5 in *F. novicida* LPS sensing could be demonstrated upon ectopic expression. (**a**,**b**) Expression profile of caspase-5 by (**a**) qRT-PCR and (**b**) Western blot analysis in hMDMs infected with wild-type *F. novicida* (WT) or the ΔFPI mutant at a MOI of 10 for 8 h. (**c**-**h**) U937 *CASP4*$^{em/em}$ cells transduced with an empty vector (p*Empty*) or a vector encoding 3xFlag-*CASP5* were infected with *F. novicida* or electroporated with 5 µg of LPS from *F. novicida* (*F.n.*) or *E. coli* (*E.c.*). (**c**) Over-expression of Caspase-5 was observed by Western blot. (**d-f**) IFN-γ-treated, PMA-differentiated U937 (Ctrl) and U937 *CASP4*$^{em/em}$ transduced with an empty vector (p*Empty*) or a vector encoding 3xFlag-*CASP5* were infected with *F. novicida* at a MOI of 100 and monitored for inflammasome activation by measuring (**d**) propidium iodide (PI) incorporation/fluorescence in real time (AU: arbitrary units), (**e**) IL-1β and (**f**) TNF release at 6 h post-infection. (**g**) Cell death was analysed by LDH assay at 4 h post-electroporation. WT U937 cells (Ctrl) transduced with empty vector were used as a positif control. (**h**) IL-1β level was quantified by ELISA from the supernatant of PMA-treated cells at 4 h post-electroporation. (**a**) Mean values ± SD from three independent experiments are shown. (**b**) One experiment representative of two independent experiments is shown. (**d**-**f**, **h**) One experiment representative of three independent experiments is shown. (**g**) Mean values ± SD from four independent experiments are shown.

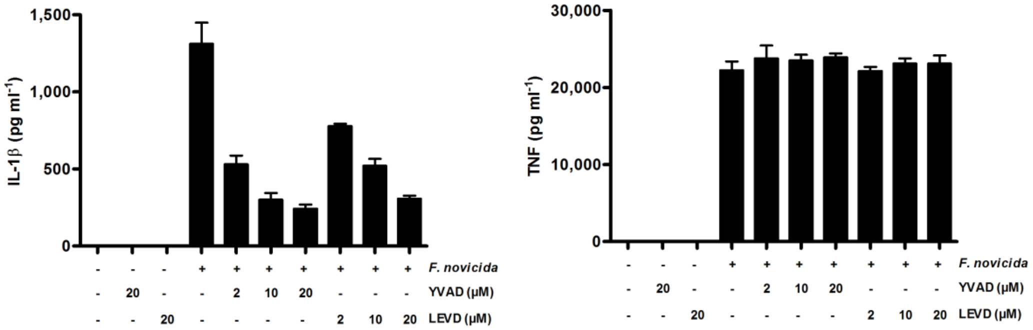

Supplementary Figure 6. Inflammatory caspase inhibitors decrease IL-1β release upon *F. novicida* infection. hMDMs were treated with z-YVAD-FMK or z-LEVD-FMK at the indicated concentrations 30 minutes before infection. IL-1β and TNF levels were quantified by ELISA at 6 h post-infection. One experiment representative of three independent experiments with mean values ± SD from three technical replicates is shown.

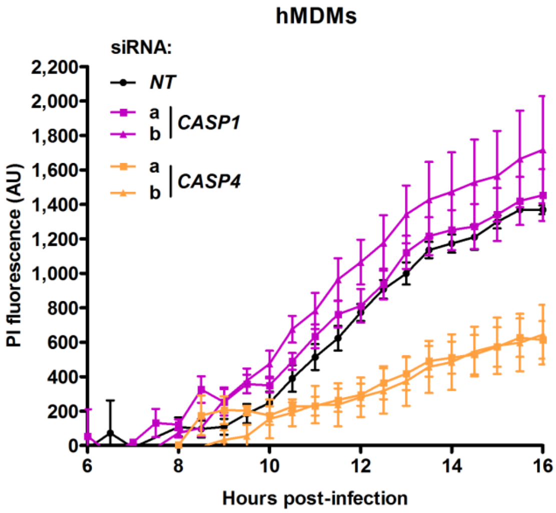

Supplementary Figure 7. Caspase-4 knockdown specifically affects hMDMs cell death kinetics upon *F. novicida* infection. hMDMs were transfected with non-targeting siRNA (NT) or two different siRNA targeting *CASP4*. 48 h post-siRNA transfection, hMDMs were infected with *F. novicida* at a MOI of 10. Cell death kinetics were determined by measuring propidium iodide (PI) incorporation/fluorescence every 30 minutes (AU : arbitrary units). Mean values ± SD from three technical replicates are shown.

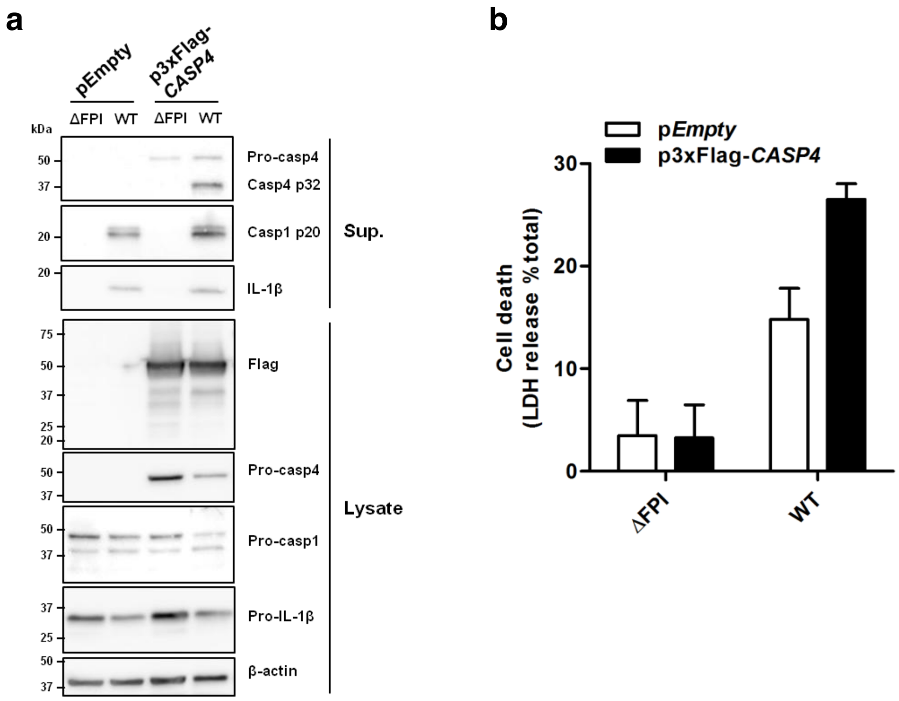

Supplementary Figure 8. Overexpression of caspase-4 allows detection of caspase-4 processing and increases cell death upon *F. novicida* infection. PMA-treated and IFN-γ-primed U937 cells transduced with an empty vector (p*Empty*) or a vector encoding 3xFlag-*CASP4* were infected with wild-type (WT) or ΔFPI *F. novicida* strains at a MOI of 100. (**a**) Caspase-4, Caspase-1 and IL-1β processing were analysed by Western blot in the supernatant (Sup.) or the lysates of infected cells at 8 h post-infection. (**b**) Cell death was analysed by LDH assay at 6 h post-infection. (**a**) One experiment representative of two independent experiments with mean values ± SD from three technical replicates is shown. (**b**) Mean values ± SD from three independent experiments are shown.

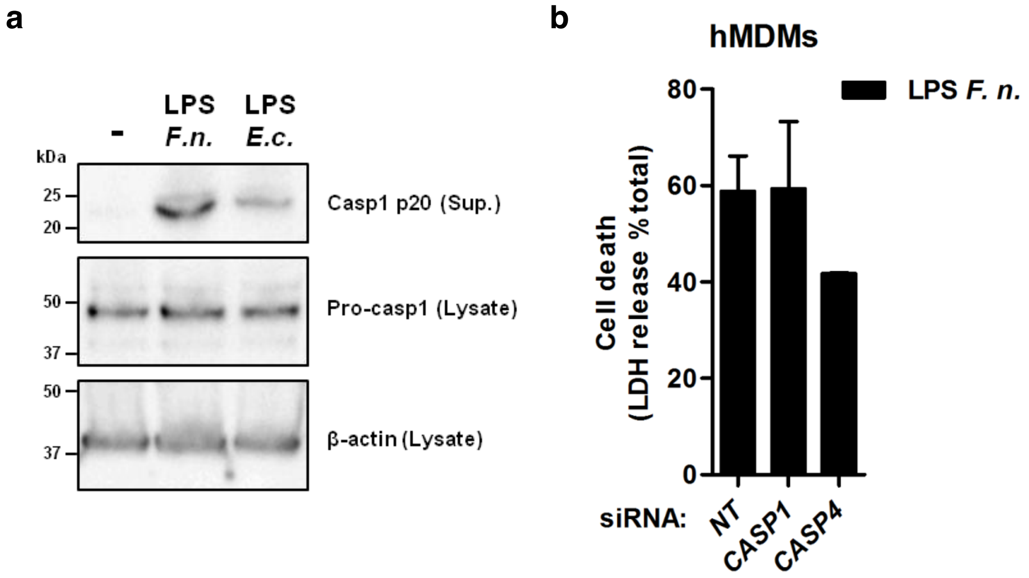

Supplementary Figure 9. ***F. novicida*** **LPS transfection triggers caspase-1 maturation and release in hMDMs. Knockdown of caspase-4 specifically decreases hMDMs cell death following** ***F. novicida*** **LPS electroporation.** (**a**) hMDMs were transfected with FugeneHD and buffer alone (-) or with 5 µg/ml of *F. novicida* (F.n.) or *E. coli* (E.c.) LPS. Caspase-1 processing/release in the supernatant was analysed by Western blot analysis at 4 h post-transfection. (**b**) hMDMs were transfected with the indicated siRNA. 48 h later, hMDMs were electroporated with 5 µg/ml of *F. novicida* LPS. Cell death was analysed by LDH release at 4 h post-electroporation. (**a**) One experiment representative of two independent experiments is shown. (**b**) Mean values ± SD from two independent experiments are shown.

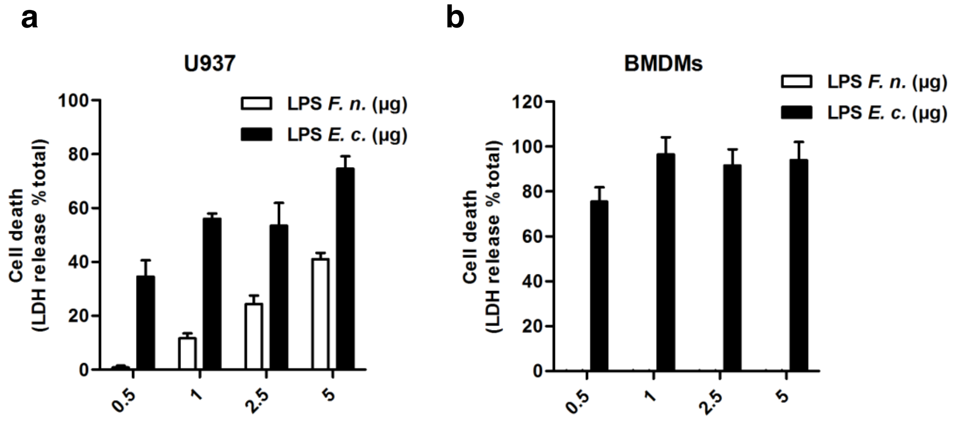

Supplementary Figure 10. ***F. novicida*** **LPS is 10-fold less potent that** ***E. coli*** **LPS to elicit cell death in human macrophages.** (**a**) U937 cells or (**b**) BMDMs were electroporated with the indicated dose of *F. novicida* (*F. n.*) or *E. coli* (*E. c.*) LPS. Cell death was quantified at 4 h post-electroporation by LDH assay. Mean values ± SD from three technical replicates are shown.

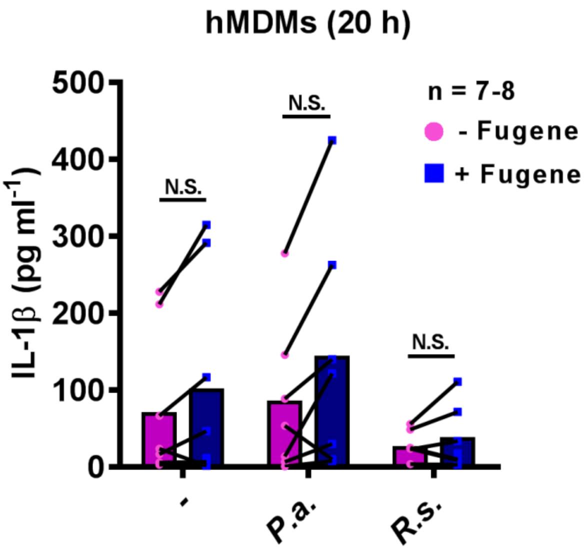

Supplementary Figure 11. Interindividual variations in the response to transfected under-acylated LPS from a *Pseudomonas aeruginosa* cystic fibrosis clinical isolate and from *Rhodobacter sphareroides* are observed. hMDMs were primed overnight with IFN-γ followed by priming with Pam3CSK4 and treatement or transfection with buffer (-) or with 5 µg/mL of LPS from a *Pseudomonas aeruginosa* cystic fibrosis clinical isolate (*P.a.*) and from *Rhodobacter sphareroides* (*R.s.*). IL-1β secretion was measured by ELISA at 20 h post-transfection. Each point shows the mean value from three technical replicates for one healthy donor. The bar shows the mean for all donors (n ≥7). The lines show the pairing of the values for a single healthy donor with or without LPS transfection (+ or - FugeneHD). N.S.: not significant by paired τ-test.

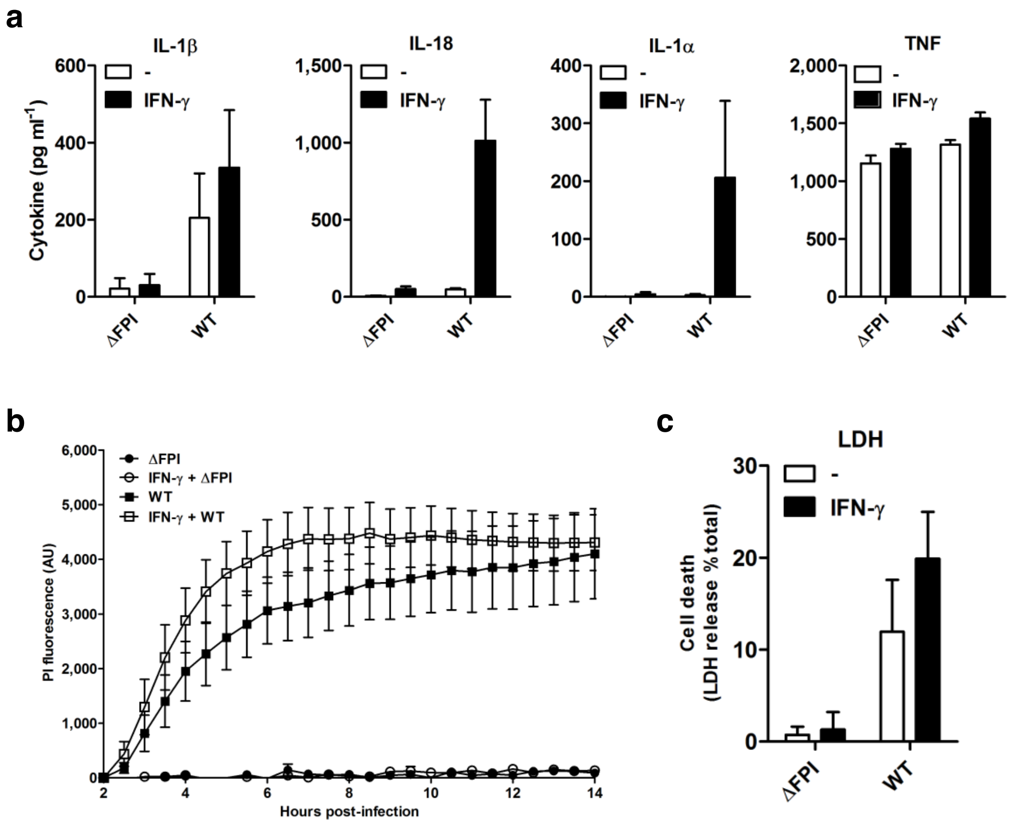

Supplementary Figure 12. IFN-γ priming enhances *F. novicida*-mediated inflammasome activation in hMDMs. (**a-c**) hMDMs were primed with IFN-γ (100 U/ml) for 16 h and then infected with wild-type *F. novicida* or the ΔFPI mutant at a MOI of 10 for 6 h. (**a**) IL-1α, IL-1β, IL-18 and TNF levels were measured by ELISA. (**b,c**) Cell death was assessed by measuring (**b**) propidium iodide (PI) incorporation/fluorescence every 30 minutes (AU: arbitrary units) and (**c**) LDH release. (**a-c**) Mean values ± SD from at least two independent experiments are shown.

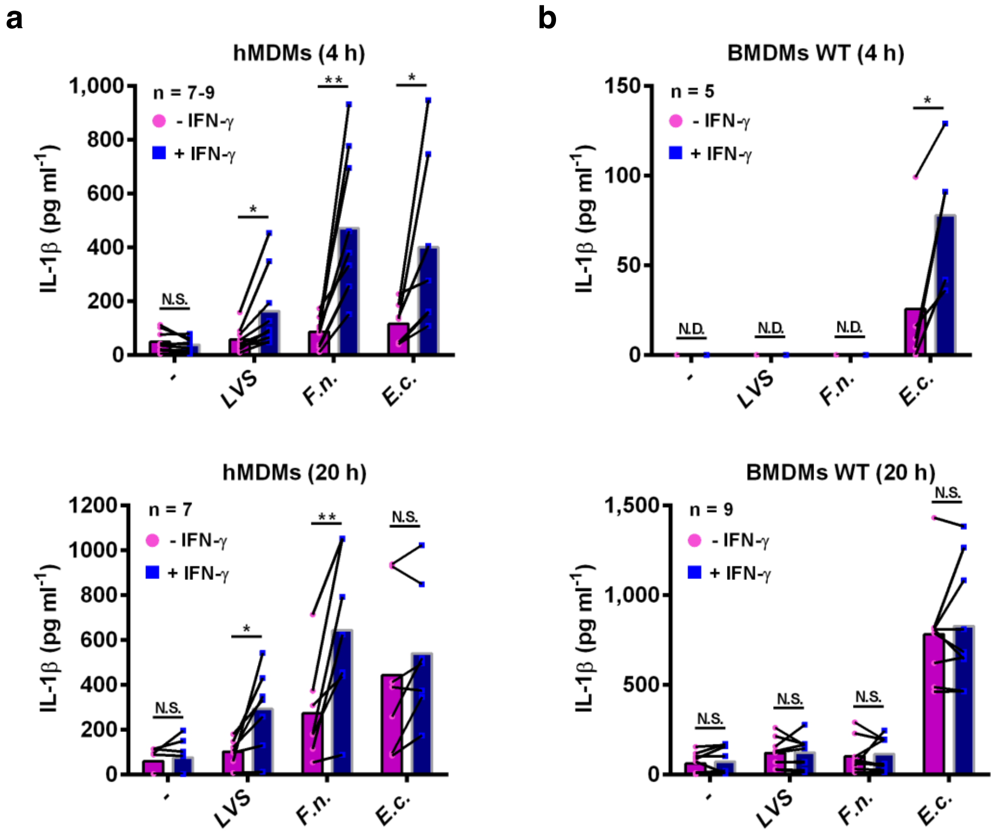

Supplementary Figure 13. IFN-γ priming enhances *F. novicida* LPS-mediated inflammasome activation in hMDMs. (**a**) hMDMs or (**b**) WT BMDMs were primed (blue) with IFN-γ for 16 h or not (magenta) followed by stimulation with Pam3CSK4 and transfected with FugeneHD alone (-) or with 5 µg/mL of LPS from *F. tularensis* live vaccine strain (*LVS*), *F. novicida* (*F.n.*), *E. coli* (*E.c.*). IL-1β levels in the supernatant were quantified by ELISA at the indicated time post-transfection. Each point shows the mean value from three technical replicates for one healthy donor. The bar shows the mean for all donors (n ≥ 5). Two-tailed p values with the following nomenclature (**P < 0.01 and *P < 0.05 by paired τ-test) are shown. N.S. Not significant. N.D. Not detected.

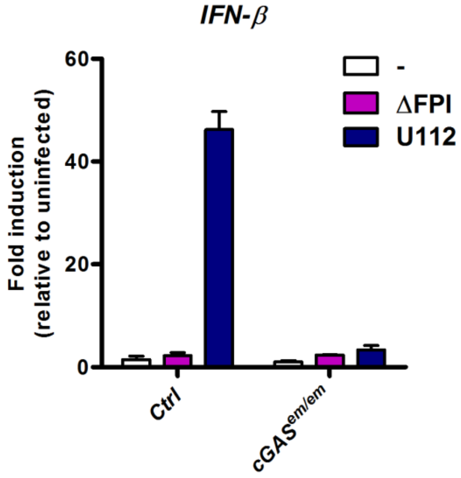

Supplementary Figure 14. ***F. novicida*** **triggers IFN-β induction through the cGAS pathway in THP-1 cells.** Quantitative RT-PCR analysis of *IFN-β* expression in PMA-treated THP-1 control (*Ctrl*) and c*GAS*$^{em/em}$ cells infected with wild-type *F. novicida* or the ΔFPI mutant at a MOI of 100 for 8 h. Mean values ± SD from three technical replicates are shown.

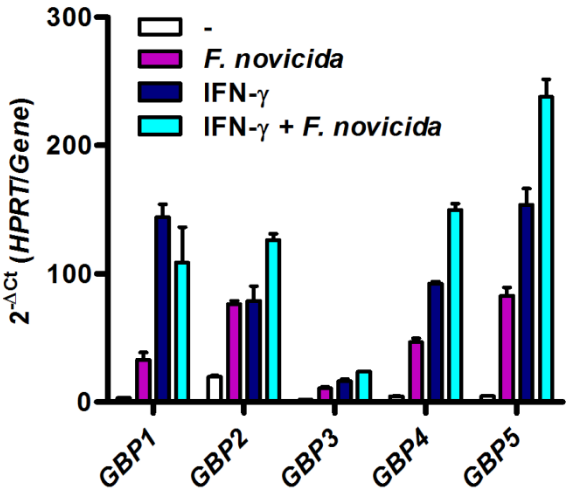

Supplementary Figure 15. IFN-γ priming increases the expression of *GBP* genes upon *F. novicida* infection. qRT-PCR analysis of *GBPs* in hMDMs after priming or not with IFN-γ and/or infection with *F. novicida* at a MOI of 10 for 8 h. One experiment representative of two independent experiments with mean values ± SD from three technical replicates is shown.

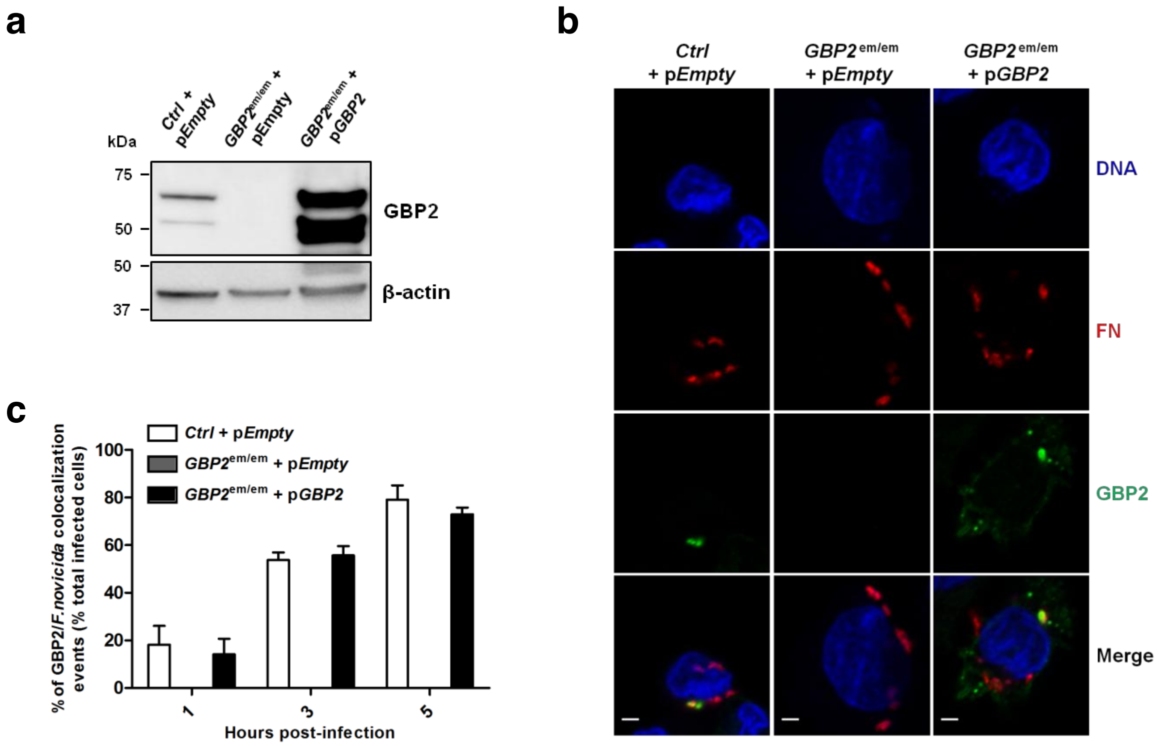

Supplementary Figure 16. GBP2 is specifically recruited to *F. novicida* in human macrophages. (**a**) *GBP2* was mutated by CRISPR/cas9 endonuclease-mediated (em) gene invalidation. The resulting cell line was validated by Western blot analysis of GBP2 expression after IFN-γ priming in U937 mock-transduced (*Ctrl* + p*Empty*), *GBP2*$^{em/em}$ U937 cells transduced with an empty vector (*GBP2*$^{em/em}$ + p*Empty*) and *GBP2*$^{em/em}$ U937 cells transduced with a lentivirus encoding GBP2 (*GBP2*$^{em/em}$ + p*GBP2*). (**b**, **c**) Analysis of GBP2/*F. novicida* colocalization by immunostaining with antibodies against *F. novicida* (FN, red), GBP2 (green) and staining with DAPI (blue) in the indicated cell lines. Cells were differentiated using PMA treatment, primed with IFN-γ and infected with *F. novicida* for 5 h. (**b**) Representative confocal images are shown. Scale bars represent 1 µm. (**c**) Quantification of colocalisation events was performed on more than 100 cells on three different coverslips per experiment. Mean values ± SD from three independent experiments are shown.

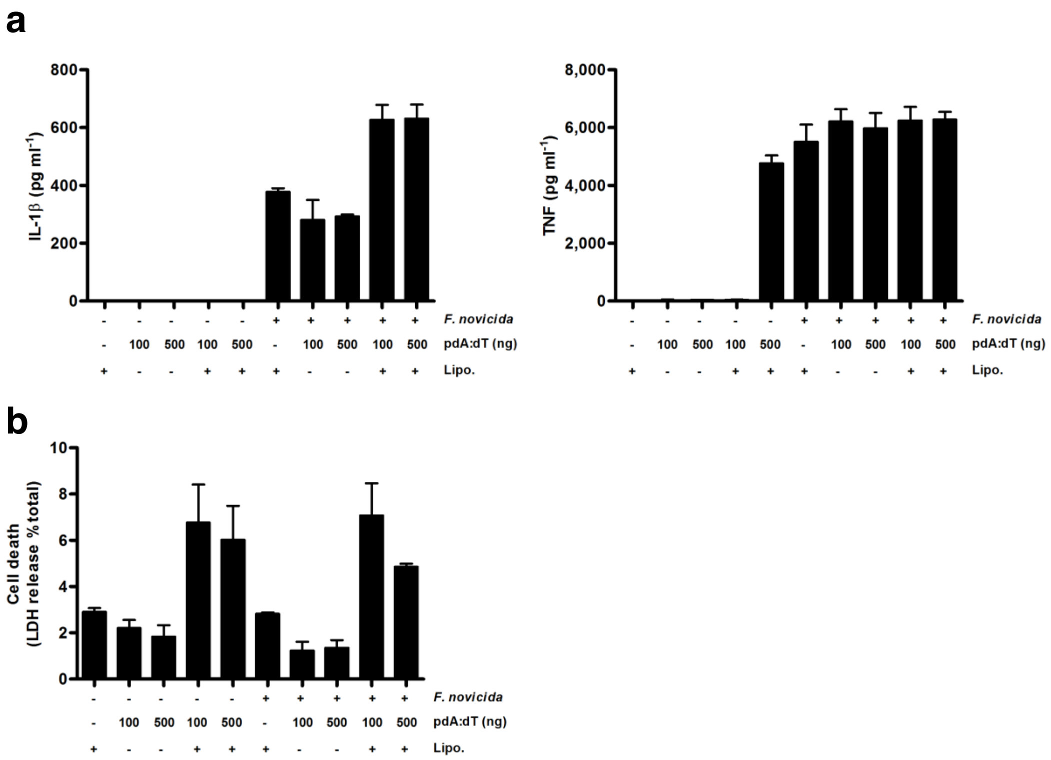

Supplementary Figure 17. ***F. novicida*** **infection does not inhibit hMDMs ability to release IL-1β or to die in response to p(dA:dT) transfection.** hMDMs were infected with *F. novicida* at a MOI of 100. At 2 h post-infection, hMDMs were transfected with poly(dA:dT). (**a**) IL-1β, TNF levels and (**b**) cell death in the supernatant were quantified by ELISA and LDH assay, respectively at 5 h post-infection (3 h post-transfection). Note that in absence of infection, no IL-1β is released due to the absence of priming. (**a,b**) One experiment representative of three independent experiments is shown. Mean values ± SD from three technical replicates are shown.

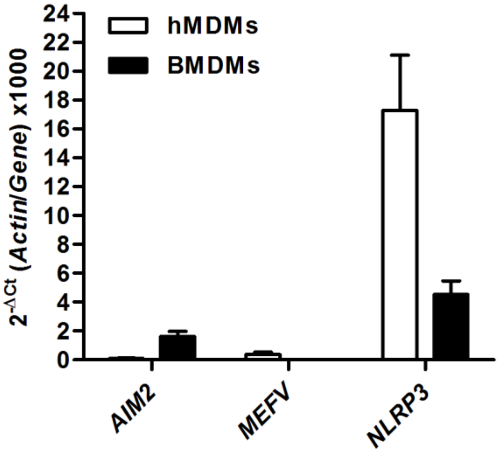

Supplementary Figure 18. Expression levels of the different inflammasome sensors differ between hMDMs and BMDMs. Expression of the indicated transcripts was determined by qRT-PCR in hMDMs or BMDMs. Mean values ± SD from two independent experiments are shown.

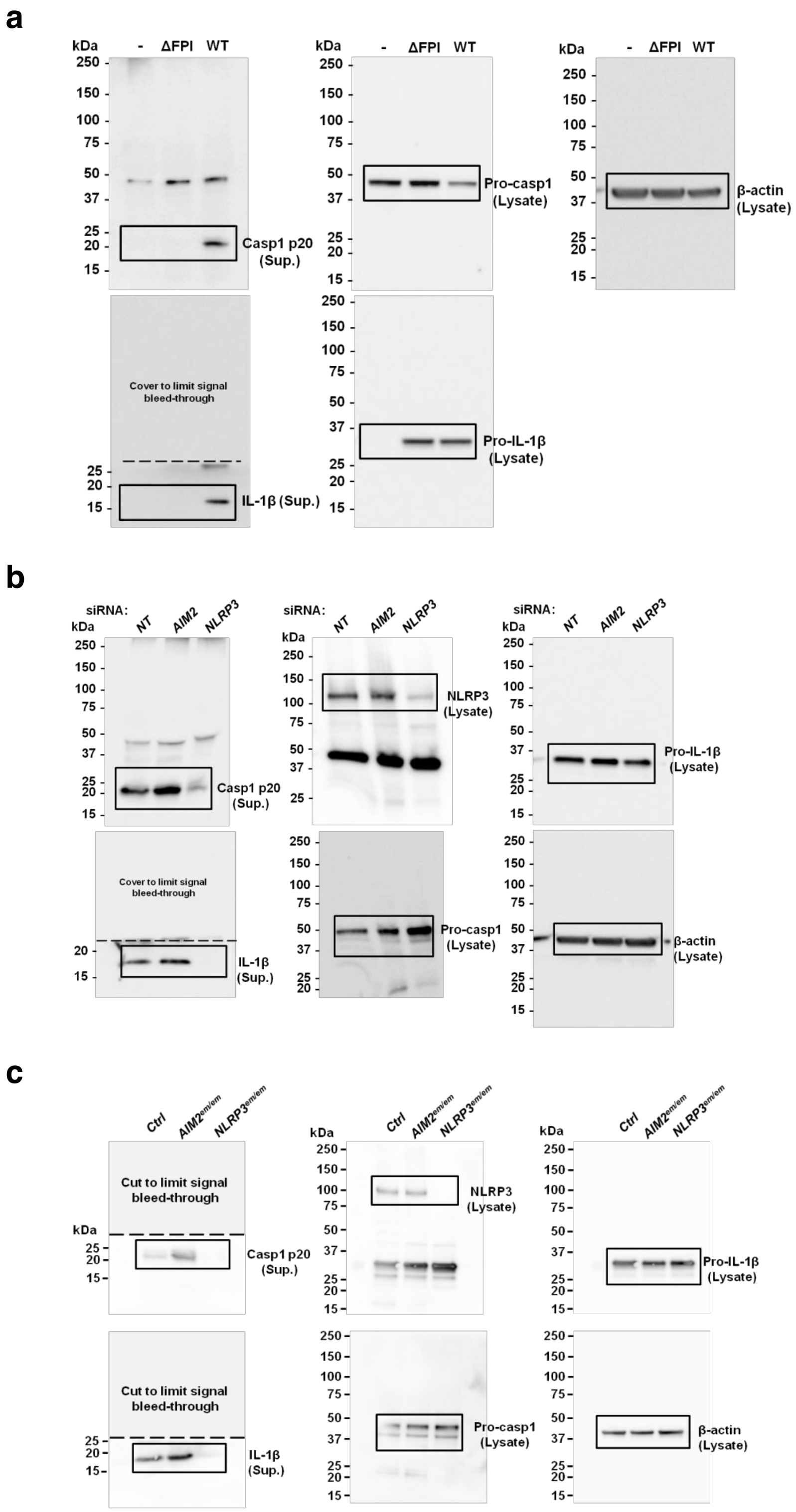
a
kDa
-
ΔFPI
WT
250
150
100
75
50
37
25
20
15
Casp1 p20 (Sup.)
Pro-casp1 (Lysate)
β-actin (Lysate)
Cover to limit signal bleed-through
IL-1β (Sup.)
Pro-IL-1β (Lysate)
b
siRNA:
NT
AIM2
NLRP3
Casp1 p20 (Sup.)
NLRP3 (Lysate)
Pro-IL-1β (Lysate)
Cover to limit signal bleed-through
IL-1β (Sup.)
Pro-casp1 (Lysate)
β-actin (Lysate)
c
Ctrl
AIM2em/em
NLRP3em/em
Cut to limit signal bleed-through
Casp1 p20 (Sup.)
NLRP3 (Lysate)
Pro-IL-1β (Lysate)
IL-1β (Sup.)
Pro-casp1 (Lysate)
β-actin (Lysate)

d

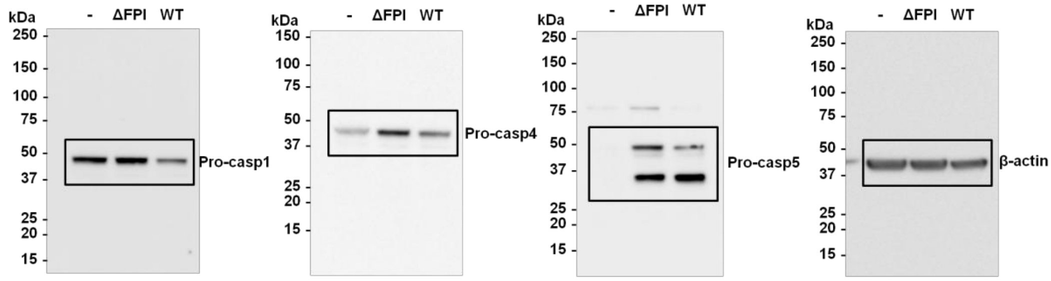

kDa
- ΔFPI WT
250
150
100
75
50
37
25
20
15
Pro-casp1
Pro-casp4
Pro-casp5
β-actin

e

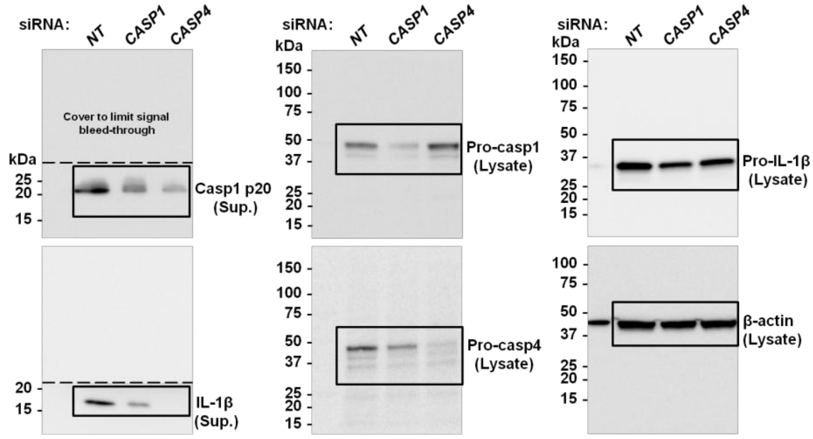

siRNA: NT CASP1 CASP4
kDa
Cover to limit signal bleed-through
Casp1 p20 (Sup.)
IL-1β (Sup.)
Pro-casp1 (Lysate)
Pro-casp4 (Lysate)
Pro-IL-1β (Lysate)
β-actin (Lysate)
150
100
75
50
37
25
20
15

f

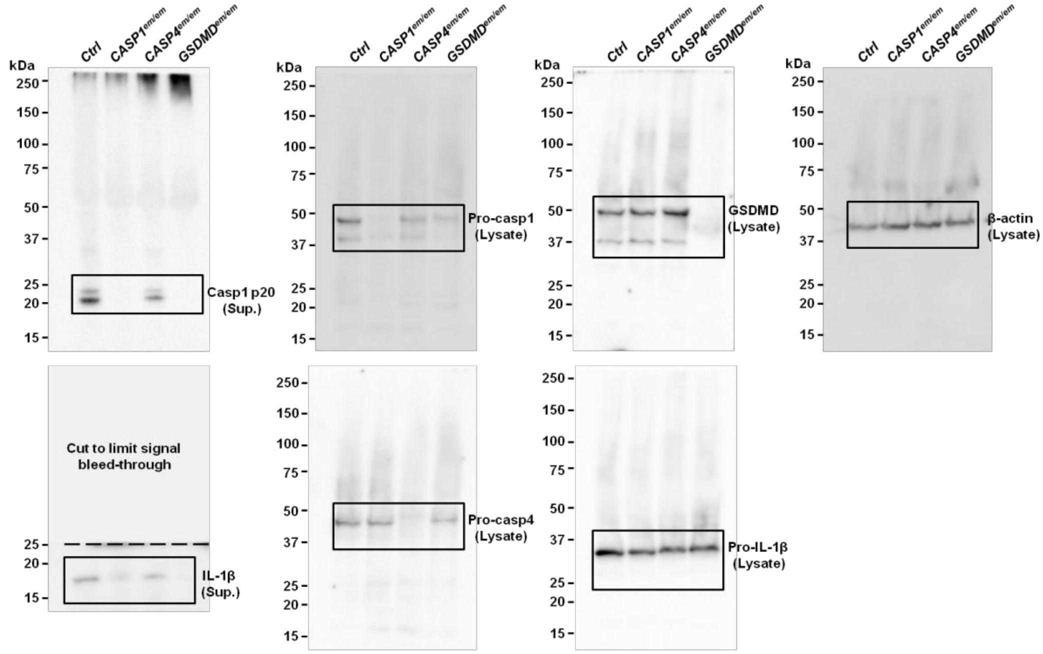

Ctrl $CASP1^{em/em}$ $CASP4^{em/em}$ $GSDMD^{em/em}$
kDa
250
150
100
75
50
37
25
20
15
Casp1 p20 (Sup.)
Pro-casp1 (Lysate)
GSDMD (Lysate)
β-actin (Lysate)
Cut to limit signal bleed-through
IL-1β (Sup.)
Pro-casp4 (Lysate)
Pro-IL-1β (Lysate)

g

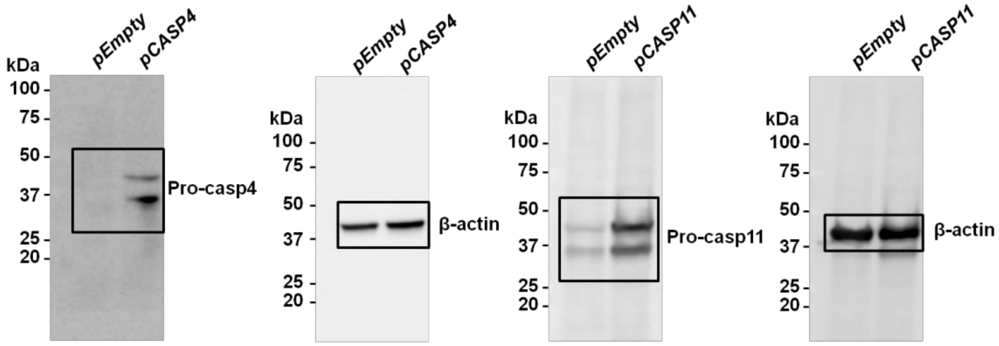
pEmpty
pCASP4
pCASP11
kDa
100
75
50
37
25
20
Pro-casp4
β-actin
Pro-casp11

h

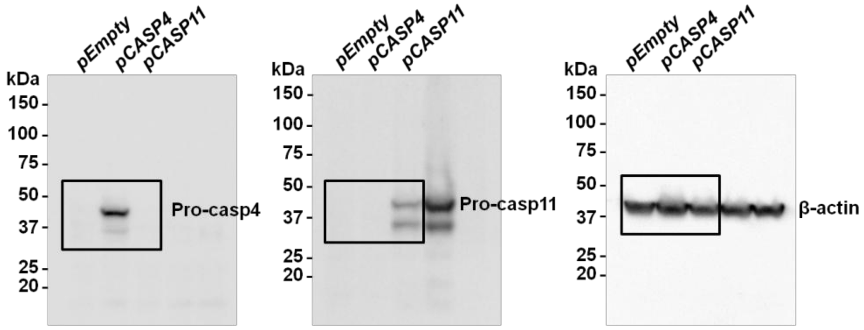
pEmpty
pCASP4
pCASP11
kDa
150
100
75
50
37
25
20
Pro-casp4
Pro-casp11
β-actin

i

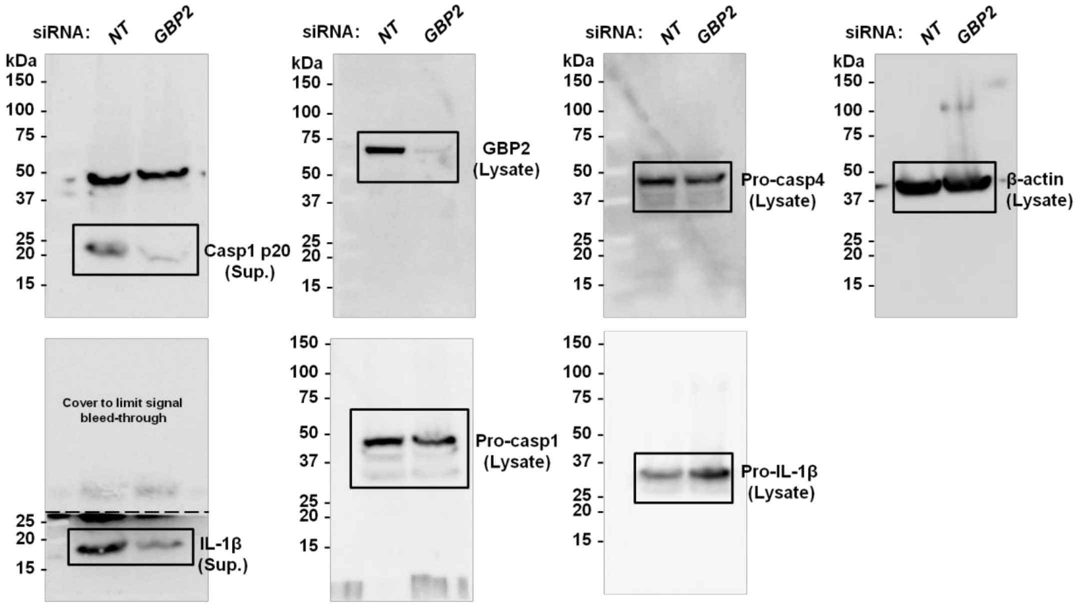
siRNA:
NT
GBP2
kDa
150
100
75
50
37
25
20
15
Casp1 p20
(Sup.)
GBP2
(Lysate)
Pro-casp4
(Lysate)
β-actin
(Lysate)
Cover to limit signal
bleed-through
IL-1β
(Sup.)
Pro-casp1
(Lysate)
Pro-IL-1β
(Lysate)

j

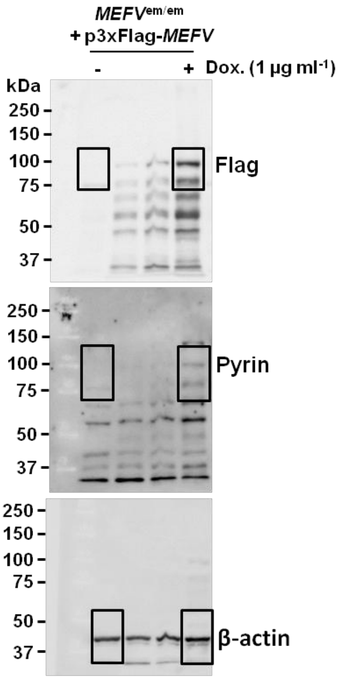

MEFV em/em
+ p3xFlag-MEFV
- + Dox. (1 µg ml-1)
kDa
250
150
100
75
50
37
Flag
Pyrin
β-actin

k

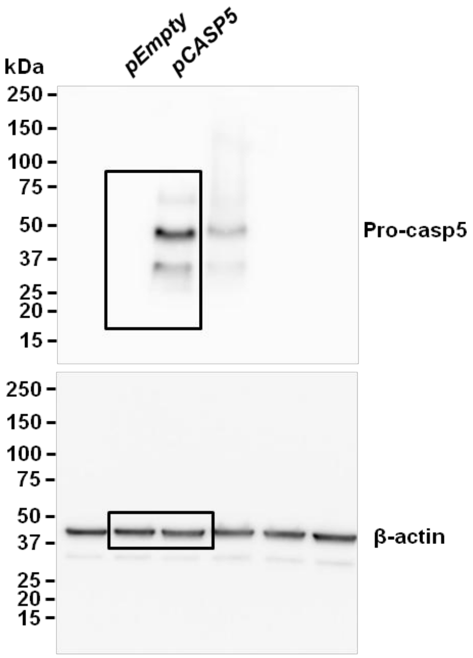

pEmpty
pCASP5
kDa
250
150
100
75
50
37
25
20
15
Pro-casp5
β-actin

l

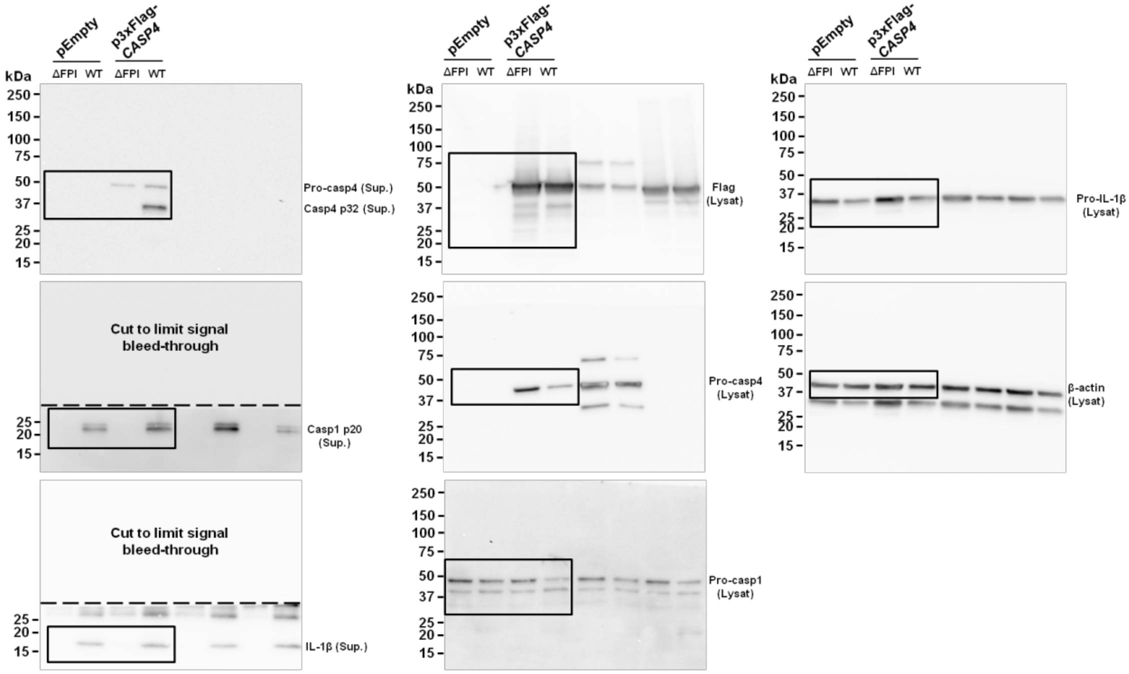

pEmpty
p3xFlag-CASP4
ΔFPI WT ΔFPI WT
kDa
250
150
100
75
50
37
25
20
15
Pro-casp4 (Sup.)
Casp4 p32 (Sup.)
Cut to limit signal bleed-through
Casp1 p20 (Sup.)
IL-1β (Sup.)
Flag (Lysat)
Pro-casp4 (Lysat)
Pro-casp1 (Lysat)
Pro-IL-1β (Lysat)
β-actin (Lysat)

m

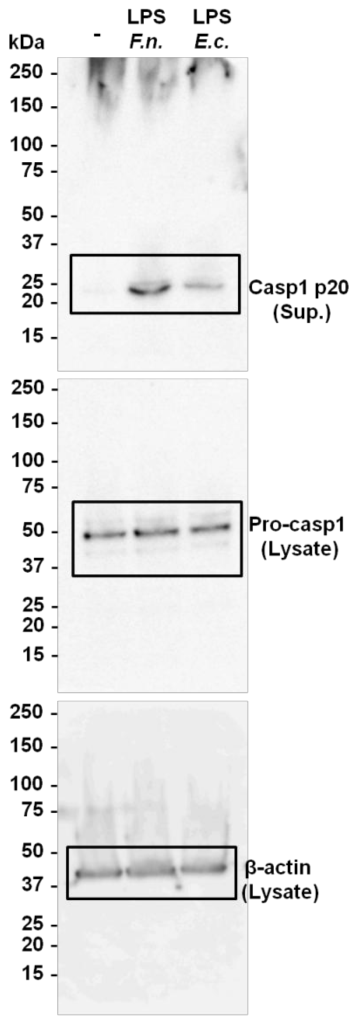

n

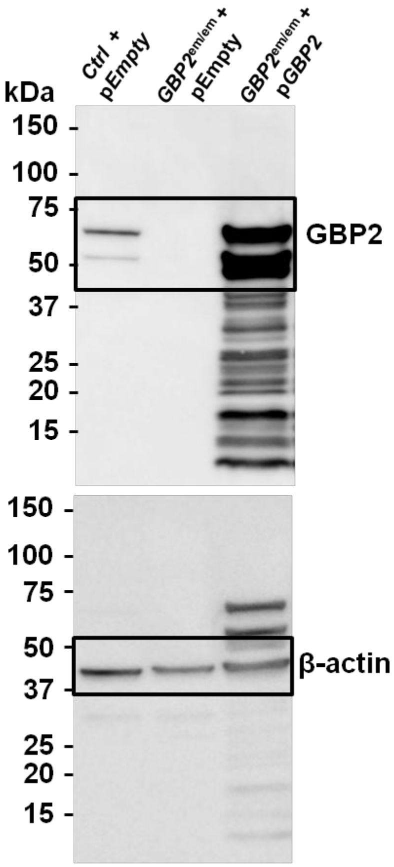

Supplementary Figure 19. Uncropped immunoblots. **(a)** Related to Fig. 1c. **(b)** Related to Fig. 2b. **(c)** Related to Fig. 2e. **(d)** Related to Fig. 3b and supplementary Fig. 5b. **(e)** Related to Fig. 3e. **(f)** Related to Fig. 3h. **(g)** Related to Fig. 5d. **(h)** Related to Fig. 5g. **(i)** Related to Fig. 8b. **(j)** Related to supplementary Fig. 3g. **(k)** Related to tsupplementary Fig. 5c. **(l)** Related to supplementary Fig. 8a. **(m)** Related to supplementary Fig. 9a. **(n)** Related to supplementary Fig. 16a. When indicated the membrane was cut or covered to limit signal bleed-through.

Supplementary Table 1: gRNA, qRT-PCR and cloning primers used in this study

CRISPR/ Cas9 primers

GBP2gRNA sens	caccggggcccggcaagttgatctc
GBP2gRNA antisens	aaacgagatcaacttgccgggcccc
NLRP3gRNA sens	caccgggatcttcgctgcgatcaac
NLRP3gRNA antisens	aaacgttgatcgcagcgaagatccc
AIM2gRNA sens 1	caccgttcacgtttgagacccaaga
AIM2gRNA antisens 1	aaactcttgggtctcaaacgtgaac
AIM2gRNA sens 2	caccgttcagcatctaacacacgtg
AIM2gRNA antisens 2	aaaccacgtgtgttagatgctga
MEFVgRNA sens 1	caccgggcgtactcttccccatagt
MEFVgRNA antisens 1	cactatggggaagagtacgcccaaa
MEFVgRNA sens 2	caccgttgaacttctcgaagtcata
MEFVgRNA antisens 2	ctatgacttcgagaagttcaacaaa
CASP1gRNA sens 1	caccacagacaagggtgctgaacagt
CASP1gRNA antisens1	taaaactgttcagcacccttgtctgt
CASP1gRNA sens 2	caccatggaaacaaaagtcggcaggt
CASP1gRNA antisens2	taaaacctgccgacttttgtttccat
CASP4gRNA sens1	caccagttatccaaaacaccagtggt
CASP4gRNA antisens1	taaaaccactggtgttttggataact
CASP4gRNA sens2	cacctgcagctcatccgaatatgggt
CASP4gRNA antisens2	taaaacccatattcggatgagctgca
GSDMDgRNA sens1	cacctgagtgtggaccctaacaccgt
GSDMDgRNA antisens1	taaaacggtgttagggtccacactc
GSDMDgRNA sens2	caccaggttgacacacttataacggt
GSDMDgRNA antisens2	taaaaccgttataagtgtgtcaacct

qRT-PCR primers

Actb-Fwd	gtggatcagcaagcaggagt
Actb-Rev	agggtgtaaaacgcagctca
Casp11-Fwd	tccagacattcttcagtgtgga
Casp11-Rev	tctggttcctccatttccag
Aim2-Fwd	agtaccgggaaatgctgt
Aim2-Rev	gcacctgcactttgaatcag
Nlrp3-Fwd	cccttggagacacaggactc
Nlrp3-Rev	gaggctgcagttgtctaattc

Mefv-Fwd	ggagatgaggggatatgtgg
Mefv-Rev	tggatttctgtttgtttcagga
HPRT-Fwd	tgaccttgatttattttgcatacc
HPRT-Rev	cgagcaagacgttcagtcct
ACTB-Fwd	attggcaatgagcggttc
ACTB-Rev	cgtggatgccacaggact
AIM2-Fwd	cagacccggtttgctgat
AIM2-Rev	ttatctccatctgacaactttgg
NLRP3-Fwd	cacctgttgtgcaatctgaag
NLRP3-Rev	gcaagatcctgacaacatgc
MEFV-Fwd	gaaatccagaacattcggtca
MEFV-Rev	taccgtcaactgggtctcct
CASP1-Fwd	ctgggactctcagcagatca
CASP1-Rev	atagctgggttgtcctgcac
CASP4-Fwd	ggcaggacaaatgcttcttc
CASP4-Rev	gacaaagcttgagggcatct
CASP5-Fwd	ttcaacaccacataacgtgtcc
CASP5-Rev	gtcaaggttgctcgttctatgg
GBP1-Fwd	ccagtgctcgtgaactaagga
GBP1-Rev	tgtcagtggatctctgatgc
GBP2-Fwd	ccctagttctgctcgacactg
GBP2-Rev	aggcaaagatccaggagtca
GBP3-Fwd	aggaaccaaggaaggggata
GBP3-Rev	aattgcatcggtcacagactc
GBP4-Fwd	gagggaatcattgtcactgga
GBP4-Rev	gatggcatctacataagtcaccac
GBP5-Fwd	cctctatcgcactggcaaat
GBP5-Rev	ctgcaccgtagatgcaacag
GBP6-Fwd	acagcatgagcaccatcaac
GBP6-Rev	ttctgtgagctccgtcacat
GBP7-Fwd	tgccttcttaccaagtccaga
GBP7-Rev	tctctgatgccatgttcagg

Cloning primers

GBP2AfeI-Fwd	attagcgctcctggacatggctccagagat
GBP2HpaI-Rev	attgttaacgggagctggacaggcaaat

CASP4BamHI-Fwd	attggatccaccatggcagaaggcaaccacagaa
CASP4NotI-Rev	attgcggccgctcaattgccaggaaagaggtaga
Casp11BamHI-Fwd	attggatccaccatggctgaaaacaaacaccctg
Casp11NotI-Rev	attgcggccgctcagttgccaggaaagaggtag
CASP4NotI-Fwd	aaaagcggccgcggcagaaggcaaccacagaaaaaag
CASP4XhoI-Rev	ttttctcgagtcaattgccaggaaagaggtag
CASP5NotI-Fwd	aaaagcggccgcgttcaaaggtatccttcagagtgg
CASP5XhoI-Rev	ttttctcgagtcaattgccaggaaagaggtagaaatc
